# Evaluation of a Telehealth-Enabled Pilot Program to Address Intensive Care Unit Health Care Worker Mental Health Distress

**DOI:** 10.1089/tmr.2023.0030

**Published:** 2023-08-04

**Authors:** Nihar Shah, Andrew J. Goodwin, Rebecca Verdin, John T. Clark, Alyssa A. Rheingold, Kenneth J. Ruggiero, Annie N. Simpson, Dee W. Ford

**Affiliations:** ^1^ Division of Pulmonary Critical Care Allergy and Sleep Medicine, Medical University of South Carolina, Charleston, South Carolina, USA.; ^2^ Telehealth Center of Excellence; Leadership and Management; Medical University of South Carolina, Charleston, South Carolina, USA.; ^3^ Biomedical Informatics Center; Leadership and Management; Medical University of South Carolina, Charleston, South Carolina, USA.; ^4^ Department of Psychiatry and Behavioral Sciences; Leadership and Management; Medical University of South Carolina, Charleston, South Carolina, USA.; ^5^ College of Nursing, Leadership and Management; Medical University of South Carolina, Charleston, South Carolina, USA.; ^6^ Department of Healthcare, Leadership and Management; Medical University of South Carolina, Charleston, South Carolina, USA.

**Keywords:** burnout, telehealth, ICU, COVID-19

## Abstract

**Introduction:**

Health care workers (HCWs) are at heightened risk of adverse mental health events (AMHEs) and burnout with resultant impact on health care staffing, outcomes, and costs. We piloted a telehealth-enabled mental health screening and support platform among HCWs in the intensive care unit (ICU) setting at a tertiary care center.

**Methods:**

A survey consisting of validated screening tools was electronically disseminated to a potential cohort of 178 ICU HCWs. Participants were given real-time feedback on their results and those at risk were provided invitations to meet with resiliency clinicians. Participants were further invited to engage in a 3-month longitudinal assessment of their well-being through repeat surveys and a weekly text-based check-in coupled with self-help tips. Programmatic engagement was evaluated and associations between at-risk scores and engagement were assessed. Qualitative input regarding programmatic uptake and acceptance was gathered through key informant interviews.

**Results:**

Fifty (28%) HCWs participated in the program. Half of the participants identified as female, and most participants were white (74%) and under the age of 50 years (93%). Nurses (38%), physicians-in-training (24%), and faculty-level physicians (20%) engaged most frequently. There were 19 (38%) requests for an appointment with a resiliency clinician. The incidence of clinically significant symptoms of AMHEs and burnout was high but not clearly associated with engagement. Additional programmatic tailoring was encouraged by key informants while time was identified as a barrier to program engagement.

**Discussion:**

A telehealth-enabled platform is a feasible approach to screening at-risk HCWs for AMHEs and can facilitate engagement with support services.

## Introduction

Health care workers (HCWs) are at heightened risk of experiencing adverse mental health events (AMHEs) such as depression, anxiety, post-traumatic stress disorder (PTSD), and burnout.^1–5^ These HCW issues contribute to adverse patient outcomes, high rates of HCW attrition, and resultant economic costs ranging between $2.6 and $6.3 billion annually in the United States.^6–9^ The COVID-19 pandemic further amplified rates of AMHEs.^10^

Concerns over personal safety, moral distress from patients dying without family present, staffing, and resource shortages, and uncertainty around frequently shifting guidance have contributed to alarming strain on health care systems.^11–16^ Recent studies have observed ongoing elevated rates of AMHEs among HCWs, yet, despite this heightened awareness, the health care industry has struggled to support its at-risk workers. Thus, programs that can identify HCW distress from AMHEs in real-time and provide them timely access to support services are sorely needed.

Prior efforts to mitigate AMHEs and promote wellness among HCWs have failed to identify a consensus approach.^17^ In addition to interventions centered on social support, positive attitude, training/education, and protective behaviors, utilization of telehealth to facilitate access to mental health services has been proposed as a potentially effective strategy.^18^ Telepsychiatry has been employed to expand the reach of mental health care for years and experienced broad uptake during the COVID-19 pandemic.^19–24^

Telehealth has also been successfully leveraged as part of screening and treatment programs targeted to trauma survivors in need of support for PTSD and other psychiatric sequelae.^24^ More recently, efforts to deliver mental health services to HCWs have increasingly utilized telehealth, particularly in the context of the pandemic.^25,26^

We sought to incorporate these emerging trends into an institutional effort to address HCW AMHEs by leveraging the substantial telehealth capabilities and expertise available at the Medical University of South Carolina (MUSC), a federally designated Telehealth Center of Excellence (HRSA U66RH31458). We designed a telehealth-enabled, automated screening, and support platform intended to (1) provide receptive HCW screening for AMHEs with real-time tailored feedback, (2) offer HCW's timely access to support services through telehealth, and (3) perform longitudinal check-ins with wellness tips.

A pilot feasibility evaluation was performed to assess uptake and engagement of the program and to elicit feedback for program refinement. HCWs in the intensive care unit (ICU) setting were targeted in the pilot program as they were anticipated to have the highest need for AMHE support.^27–30^ Herein, we report the findings of this pilot program to aid future efforts to provide needed support to HCWs.

## Methodology

### Study setting and target population

This study was performed at MUSC, a quaternary care academic medical center in the southeastern United States. This study was determined to be quality improvement by the MUSC institutional review board and exempted from review. HCWs in two, predominantly medical, ICUs were selected for the pilot study based on perceived unmet needs in the context of the COVID-19 pandemic and resultant staffing shortages. The HCW cohort consisted of registered nurses (RNs), respiratory therapists (RTs), patient care technologists (PCTs), pharmacists, advanced practice providers (APPs), and physicians including internal medicine residents, pulmonary and critical care fellows, and pulmonary and critical care faculty with a potential cohort size of 178.

### Assessment tool development

A multidisciplinary team comprising clinical and behavioral psychologists, critical care faculty, and fellows reviewed relevant literature and selected complete or subdomains of validated tools to screen for AMHEs including the 9-item patient health questionnaire (PHQ-9, major depressive disorder); the 7-item generalized anxiety disorder (GAD-7); the abridged 8-item post-traumatic stress disorder checklist for *DSM-5* (PCL-5); the Patient-Reported Outcomes Measurement Information System (PROMIS, sleep disturbance); the Oldenburg Burnout Inventory (OBI); the alcohol use disorders identification test (AUDIT-C); and the 10-item Connor–Davidson Resilience Scale.^10,31–45^

Additional detail regarding these tools are given in Supplementary Table S1. The initial screening questionnaire comprised 71 questions including the 61 questions of the above tools and 10 additional demographic questions including age, gender, race and ethnicity, discipline, years of experience, and whether the respondent works in defined shifts.

Once the initial screening questionnaire was finalized, six ICU stakeholders including three ICU RNs, one RT, and two physicians were invited to voluntarily beta test the survey to provide feedback on survey length, pertinence of questions to ICU HCWs, and likelihood to engage in the program. Feedback was obtained through a four-question survey and through qualitative field notes (Supplementary Table S2).

### Clinical reasoning/process mapping

**FIG. 1.**
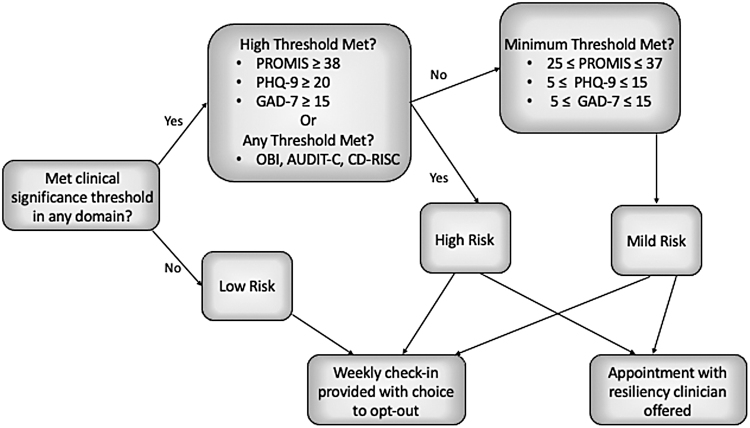
Participants were stratified into low-, mild-, and high-risk categories using validated thresholds for screening tools and offered weekly check-ins as well as appointments with resiliency clinicians for those at risk.

Before distributing the assessment tool to the targeted cohort of ICU HCWs, we developed a process map of how the program would score and give feedback to participants (Fig. 1). We utilized the known clinically significant thresholds of the selected tools to stratify respondents into low-, mild-, and high-risk groups (Supplementary Materials). If no clinically significant threshold was reached, respondents were categorized as low risk. If a respondent exceeded a threshold for mild risk on any tool that included such a threshold (e.g., PHQ-9, GAD-7, and PROMIS), then he or she was categorized as mild risk.

If a respondent exceeded the high-risk threshold on the PHQ-9, GAD-7, or PROMIS tools or exceeded the clinically significant threshold on any of the other tools, then they were categorized as high risk. Respondents received immediate tailored feedback regarding their risk category (Supplementary Table S3). Any respondent who was categorized as either mild or high risk was offered the telephone number and e-mail contact to schedule an appointment with an MUSC resiliency clinician who was not a part of the research team.

If any respondent answered the PHQ-9 question: “Over the past 2 weeks, how often have you had thoughts that you would be better off dead or of hurting yourself in some way?” with any answer other than “not at all,” the platform immediately responded in two ways. First, the platform provided the participants with the telephone number to the national suicide hotline and recommended that they call 911 or go to the nearest emergency room if they were at risk for harming themselves. Second, the platform generated an alert e-mail to an MUSC resiliency clinician who then contacted the participant directly.

### Longitudinal tracking

Respondents were also given the opportunity to engage with the program longitudinally through weekly check-ins that were delivered through cell phone text message. These texts provided a link to the Kessler Psychological Distress Scale (K6)^46,47^ through a Research Electronic Data Capture (REDCap™) survey link. Respondents were again given immediate tailored feedback (Supplementary Table S4) based on their score including the referral number to the MUSC Resiliency Program if survey scores indicated at least moderate distress.

In addition, respondents of the weekly check-in were provided a stress relieving tip of the week (Supplementary Table S5) with a link to an associated video describing that tip. Respondents were given the opportunity to opt out of the weekly check-ins through text response.

Finally, all respondents of the initial screening who did not opt out of the program received the complete 61-question screening tool again at 30, 60, and 90 days of the program to assess longitudinal trends in AMHEs as well as willingness to engage in repeated assessments of well-being.

### Platform build and distribution

After the screening tools and process map were finalized, they were built into REDCap surveys using branching logic statements to provide the tailored feedback. A field was included to capture respondents' cellular telephone numbers to facilitate text-based distribution of weekly check-ins. The comprehensive 71-item survey was distributed in June 2022 to the target population through e-mail list-serves (Supplementary Table S6) as an opportunity to participate in a 3-month program focused on burnout and resiliency. Two e-mail reminders were distributed weekly to HCWs who had not already engaged in the program.

**FIG. 2.**
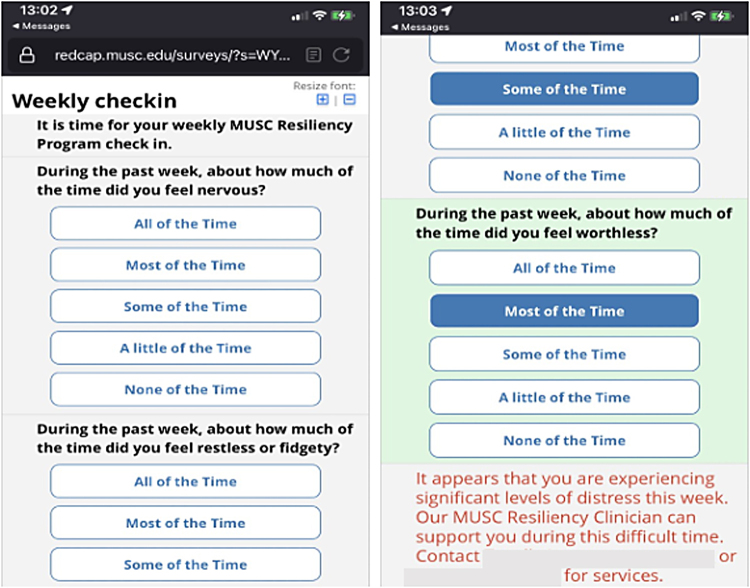
Screenshot of a weekly check-in constructed in REDCap™ and disseminated utilizing Twilio.

Twilio, a third-party web service that integrates with REDCap, was utilized to provide personalized automated feedback after completion of the initial assessment and to disseminate the invitations to participate in the weekly check-in and weekly self-help tips and videos (Fig. 2). Respondents were incentivized with $10 e-gift cards upon completion of the initial and 90-day survey. Telehealth visits were scheduled for respondents expressing interest in a 1:1 meeting with a resiliency clinician.

### Qualitative assessment

After conclusion of the study period, we sought qualitative feedback through a structured interview comprising open-ended questions authored by our multidisciplinary team to gauge participants' perceptions and critique regarding the multifaceted domains of our pilot program. All initial participants were invited to take part and up to three invitation e-mails were sent. Seven respondents were interviewed 1:1 either in person or using a videoconferencing platform in the presence of the interviewer and a second transcriber. All responses were transcribed independently by two team members for review, and an iterative approach to thematic analysis was utilized to recognize barriers to program uptake, positive or negative critique, and suggested improvements.

### Statistical analysis

Respondent demographics and screener response data are presented using descriptive statistics. Respondents were dichotomized as “more engaged” or “less engaged” with the monthly screener component of the platform if they completed ≥1 or 0 of the monthly longitudinal screeners, respectively. Similarly, respondents were dichotomized as “more engaged” or “less engaged” with the weekly check-in component of the platform if they completed ≥4 or <4 of the 12 available weekly check-ins, respectively.

The scores on each domain of the baseline screener were compared between the more engaged and less engaged subgroups using two-sided *t*-tests after the scores were found to be normally distributed. Chi square test was performed to test for associations between demographics and engagement subgrouping. All analyses were performed in SAS, version 9.4 (Cary, NC).

## Results

Of the planned potential cohort of 178, 50 (28%) HCWs chose to participate in the program. Their demographics are displayed in Table 1. Half of the participants (50%) identified as female, and most participants were white (74%) and under the age of 50 years (93%). Nurses represented a plurality of participants (38%) followed by physicians-in-training (24%) and faculty-level physicians (20%).

**Table 1.** Respondent Characteristics

Demographic	Subjects (%) ***n*** = 50
Gender	
Male	19 (38)
Female	25 (50)
Not reported	6 (12)
Age group (years)	
18–35	27 (61)
36–50	14 (32)
51–65	3 (7)
>65	0 (0)
Race	
White	37 (74)
Black	3 (6)
American Indian or Alaskan Native	1 (2)
Asian	6 (12)
Native Hawaiian or Pacific Islander	0
Not reported	3 (6)
Ethnicity	
Hispanic	1 (2)
Not reported	7 (14)
Discipline	
Intensive care unit nurse	19 (38)
Pulmonary and critical care faculty	10 (20)
Internal medicine resident	6 (12)
Pulmonary and critical care fellow	6 (12)
Patient care technologist	2 (4)
Other	1 (2)
Years in discipline	
0–5	14 (28)
5–15	11 (22)
>15	3 (6)
Not reported	22 (44)

### Programmatic uptake and longitudinal engagement

From the 50 participants, there were 19 requests for an appointment with a resiliency clinician. To protect the privacy of participants, data were not captured regarding appointment attendance, clinical recommendations from, or impact of any appointments. Longitudinal engagement with the program was assessed based on willingness to participate in (1) serial assessments of the complete screening tool that were administered at days 30, 60, and 90 of the programs and (2) weekly check-ins delivered through text messaging.

**FIG. 3.**
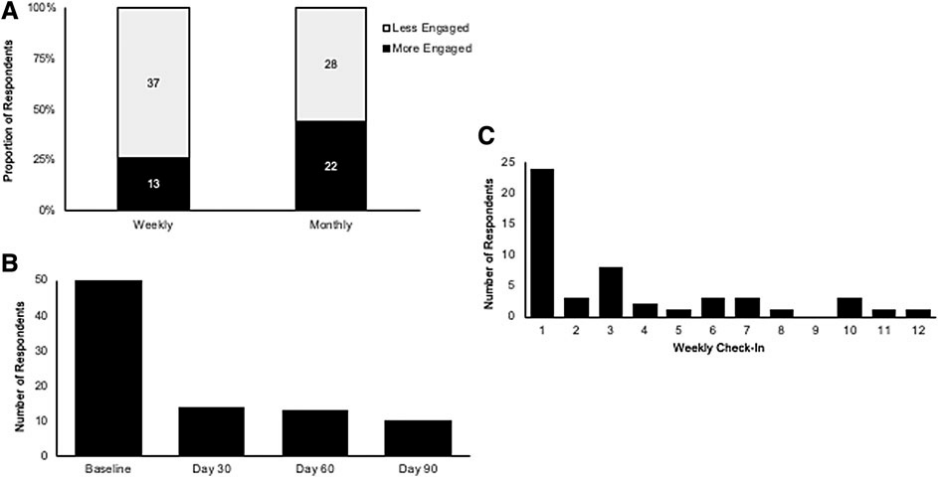
Overall, participants demonstrated modest engagement in weekly and monthly longitudinal assessments **(A)**. Longitudinal engagement with monthly screeners after baseline assessment was consistent over time **(B)** whereas engagement with weekly check-ins decreased after the first week **(C)**.

Twenty-two (44%) participants were classified as “more engaged” based on their participation with at least one of the serial screens, whereas 13 (26%) participants were deemed “more engaged” because of a willingness to participate in at least 4 weekly check-ins (Fig. 3A). Although nearly half of participants were willing to take at least one serial screening assessment, the mean number of participants who filled out each monthly screener was modest (Fig. 3B). Similarly, although participation in the first weekly check-in was higher (48%), participation in subsequent weeks was variable but overall low (Fig. 3C).

### Prevalence of AMHEs among program participants

Scores from the baseline screening tool were used to estimate the prevalence of burnout and AMHEs among HCWs who were willing to engage with the program. For tools with more than one clinically significant threshold, any score above the milder threshold was considered potentially clinically significant. More than half of program participants exceeded clinically significant thresholds for burnout, resilience, and alcohol use (Table 2). In addition, at least one-third of participants reported clinically significant symptoms of depression, anxiety, and sleep disturbance.

**Table 2.** Prevalence of Adverse Mental Health Events/Burnout

Domain	Tool (***N*** respondents)	Minimum clinical significance threshold	Median score (IQR)	Respondents above threshold (%)
Sleep disturbance	PROMIS (50)	25	24 (10–38)	38
Depression	PHQ-9 (48)	5	5 (0–18)	38
Anxiety	GAD-7 (49)	5	4 (0–20)	33
Post-traumatic stress disorder	PCL5 (46)	31	7.5 (0–55)	11
Resiliency	CD-RISC10 (46)	32	30 (17–40)	50
Alcohol use	AUDIT-C (38)	3	3 (1–12)	60
Burnout	OBI (disengagement) (45)	2.1	2.13 (1–3.25)	55
Burnout	OBI (exhaustion) (45)	2.25	2.63 (1–3.75)	58

AUDIT-C, alcohol use disorders identification test; PHQ-9, 9-item patient health questionnaire; GAD-7, 7-item generalized anxiety disorder; PROMIS, Patient-Reported Outcomes Measurement Information System; OBI, Oldenburg Burnout Inventory; PCL-5, Post-traumatic Stress Disorder Checklist for *DSM-5*; CD-RISC10, 10-item Connor–Davidson Resilience Scale.

In univariate testing of AMHE scores and participation with the program, we found no associations between demographics or baseline scores in any domain and being engaged with the program (data not shown). There was insufficient power to assess longitudinal changes in scores and their potential relationship with baseline scores or engagement status (data not shown).

### Qualitative assessment

After conclusion of the pilot program, we interviewed seven participants (six physicians and one APP) who volunteered to provide qualitative feedback regarding perceived strengths and weaknesses of the program as well as proposed refinements. We analyzed these interviews to identify motivators and detractors impacting ongoing engagement and utilization of services offered by the program. These are summarized with illustrative quotes in Table 3. Multiple interviewees found the program's positive reinforcement and ability to provoke self-insight as positive motivators while several acknowledged that time limitations amid a busy schedule combined with the frequency of check-ins and screenings hindered longitudinal engagement.

**Table 3.** Qualitative Assessment

Theme	Representative comment
Motivators	
Insight provoking	“Reminder to be mindful of mental health and how to approach different things in life”
Positive reinforcement	“Helped refocus on things I was already aware of. For example, getting enough sleep but making sure it is more of a priority”
Viewed as a coping tool	“Feedback was helpful, allows you to cope with things you might not feel comfortable with discussing in public in an anonymous way”
Detractors	
Limitation of time	“Time biggest constraint; was in school and working”
Multiple check-ins/questionnaires	“Remember getting text but was at a point where he was really busy and didn't feel like he was going to score any differently like he had before”
Pervasive culture of shame in seeking help	“Pervasive culture in medicine that promotes can handle myself, I'm fine”
Recommendations	
Desire for tailored feedback	“Only recommendation would be to make it more personal, ID areas person struggling with and then focus the follow-ups on those areas rather than generalized”
Less frequent check-ins	“Occurring too frequently so regularly ignored”
Extend to additional health care workers	“Really like the program and wish the program can be extended to other specialties and other healthcare workers”

Several interviewees were hopeful that the program could be expanded to other HCWs but also recommended tailoring of both the longitudinal check-ins and the provided self-help tips based on which domains were clinically relevant on a participant's baseline screening.

## Discussion

HCWs experience high rates of AMHEs, an epidemic made worse by the COVID-19 pandemic crisis. However, health systems struggle with timely identification of these issues and, more importantly, with timely connection of affected HCWs with support. In this pilot program, we demonstrated that ICU HCWs had a high degree of willingness to participate in a telehealth-enabled program intended to screen for AMHEs, connect HCWs to support services, and provide self-help resources. We used an inclusive approach to targeting all ICU HCWs including RTs and PCTs due to the high impact of burnout across all disciplines as well as observed staff attrition among all ICU care team members.^48,49^

There were additional salient findings in this pilot program that suggest the potential value of this program. The participant population demonstrated a high prevalence of AMHEs, like previous reports^10,30,50^ confirming an ongoing need for HCW support. Program participants also demonstrated a willingness to connect with resiliency support services and appreciated the program's ability to generate self-insight. However, despite these positive features, longitudinal engagement was modest that limited the ability to assess changes in AMHE prevalence over the course of the program.

The telehealth-centered design of the platform provided several potential benefits that could translate into improved support for HCWs. First, incorporation of automated text links to assessments and self-help resources expanded the program's reach and offered greater convenience of completing assessments quickly and from anywhere. In addition, the platform's ability to provide real-time scoring feedback coupled with simultaneous access to the contact information for a resiliency clinician enhanced the likelihood that an at-risk participant sought needed support. This was suggested by our finding that 38% of participants requested an appointment with the MUSC Resiliency Program, an uptake rate nearly half of that observed among severe trauma survivors in an analogous program.^24^

Furthermore, offering these appointments virtually through telehealth provided a convenience whereby HCWs could schedule appointments with greater flexibility and anonymity that may have contributed to program uptake. Lastly, the platform's automated infrastructure offers both scalability and adaptability allowing for easy translation to other health systems.

This pilot initiative also yielded important findings that can and will be used for programmatic refinement. Modest longitudinal engagement rates were observed that may limit the impact of the program on HCW well-being. Qualitative feedback from participants suggested several possible contributors to participant attrition. The combination of busy schedules with little spare time and the frequency of the program's check-ins were cited by multiple participants as a detractor. Furthermore, multiple participants commented that they would appreciate additional tailoring of the program to their individual needs including customization of their ongoing assessments and resources based on their initial screen results.

This feedback suggests that even if the check-ins are very brief (e.g., six multiple choice questions), weekly check-ins and monthly assessments may be too frequent for this population. However, enhancing the value of the longitudinal touches through customization could impact engagement.

Although designed as a pilot program that was not intended to be definitive, we acknowledge that this evaluation has limitations. The modest longitudinal engagement rate impeded our ability to assess for changes in AMHEs and burnout over the duration of the program. Future iterations of the program will target larger HCW populations and will include feedback-guided refinements including participant-selected frequency of check-ins, tailored/customized check-ins, and participant-selected timing of the delivery of check-ins. It is possible that the prevalence of AMHEs observed here is confounded by selection bias as only HCWs willing to participate in the program were assessed.

However, the goal of this pilot program was not to measure AMHEs and burnout rates among ICU clinicians, as this has been well established. Rather, our objective was to design and conduct a pilot feasibility evaluation of a proactive novel telehealth intervention to better meet ICU HCW specific needs. We acknowledge that this pilot program was confined to ICU clinicians who had provided a large volume of COVID-19-related care in the past 3 years and who worked in a single quaternary academic medical center. Thus, not all the findings of this pilot study may be generalizable.

## Conclusion

In conclusion, we have demonstrated in this pilot study that ICU-based HCWs are willing to participate in a telehealth-enabled program intended to identify those who are at risk and connect them with support. Future study will include program refinement to enhance participation and optimize longitudinal engagement that will allow for a more definitive assessment of the program's impact on HCW well-being.

## Supplementary Material

Supplemental data

## Abbreviations Used

AMHEs: adverse mental health events
APPs: advanced practice providers
AUDIT-C: alcohol use disorders identification test
CD-RISC10: 10-item Connor-Davidson Resilience Scale
GAD-7: 7-item generalized anxiety disorder
HCWs: health care workers
ICU: intensive care unit
MUSC: Medical University of South Carolina
OBI: Oldenburg Burnout Inventory
PCTs: patient care technologists
PHQ-9: 9-item patient health questionnaire
PROMIS: patient-reported outcomes measurement information system
PTSD: post-traumatic stress disorder
RNs: registered nurses
RTs: respiratory therapists
