# Supplementary material for: Evaluation of a Telehealth-Enabled Pilot Program to Address Intensive Care Unit Health Care Worker Mental Health Distress
Source: Telemed Rep. 2023 Aug 4;4(1):249–58. doi: 10.1089/tmr.2023.0030 (PMC10457649; doi:10.1089/tmr.2023.0030)
Supplement: Supplemental data [file Suppl_Data.docx]

| Table S1 (Screening Tools) | | | | |
| --- | --- | --- | --- | --- |
| Domain | Tool | Number of questions | Screening period | Score range |
| Sleep disturbance | PROMIS | 8 | Last 7 days | 32-73.3 |
| Depression | PHQ-9 | 9 | Last 2 weeks | 0-27 |
| Anxiety | GAD-7 | 7 | Last 2 weeks | 0-21 |
| PTSD | PCL-5 | 8 | Last 30 days | 0-80 |
| Resiliency | CD-RISC-10 | 10 | Last 30 days | 0-40 |
| Alcohol use | AUDIT-C | 3 | Last 1 year | 0-12 |
| Burnout | OBI | 16 | N/A | 16-64 |

Sleep Disturbance:

The 8-item PROMIS sleep disturbance short form was utilized to assess the domain of sleep disturbance in individuals 18 year or older. Each item in the tool asks the patient to rate the severity of the patients sleep disturbance in the last 7 days. Each item required a response on a 5-point Likert scale providing a raw score. This raw score was utilized to calculate a t-score using a validated formula. T-score from 32 to 73.3, (32–54: within normal limits, 55–60: mild, 61–70: moderate, 71–73.3: severe) measured sleep disturbance.

Major Depressive Disorder:

The 9-item Patient Health Questionnaire (PHQ-9) is a validated tool for assessment of depressive symptoms over the preceding 2 weeks. The total score ranges from 0-27 (0-4 minimal/none, 5-9 mild, 10-14 moderate, 15-19 moderately severe, 20-27 severe). It has been utilized as a screening tool amongst HCWs during the COVID-19 pandemic.

Generalized Anxiety Disorder:

The 7-item Generalized Anxiety Disorder (GAD-7) is a validated tool for assessing the severity of anxiety symptoms over the preceding 2 weeks. The total score ranges from 0-21 (5-9 mild, 10-14 moderate, >15 severe). The GAD-7 has also been utilized in similar endeavors with the aim of quantifying the severity of anxiety amongst HCWs during the COVID-19 pandemic.

PTSD:

We utilized an abbreviated 8 item PCL-5 tool with a validated conversion formula to derive a total score to study prevalence of PTSD amongst HCWs. Responses on the PCL-5 are on a 5-point Likert scale (ranging from not at all to extremely) measuring bothersome symptoms over the preceding 1 month. The cutoff for the PCL-5 ranges from 31-33 across various studies and we utilized a cutoff of 31 to increase sensitivity.

Resilience:

The CD-RISC is a tool validated to measure resilience. The 10-item CD-RISC has been validated like the original 25 item tool. Responses are recorded on a 5-point scale (0 to 4) with a score ranging from 4 to 40. It measures as a measure of hardiness measuring flexibility, sense of self-efficacy, ability to regulate emotion, optimism, cognitive focus/maintaining attention under stress over the preceding 1 month. We utilized a cut off value of 32 or greater as signifying resilience.

Alcohol Misuse:

The AUDIT-C consists of 3 questions pertaining to alcohol misuse based on individualized consumption habits. In males a score >/=4 suggests alcohol misuse while a score >/=3 in females suggests alcohol misuse.

Burnout:

We utilized the Oldenburg Burnout Inventory (OBI) which is a 16-item validated tool to assess burnout. It measures responses on a 4-point Likert scale within the domains of disengagement as well as exhaustion. We utilized a cut off score of >/=2.25 for exhaustion and >/=2.1 for disengagement. The OBI is validated for use amongst HCWs and has been utilized in large cross-sectional studies during the COVID-19 pandemic to assess burnout amongst HCWs.

| Table S2: Qualitative Feedback during Cognitive Pre-Testing | |
| --- | --- |
| Participant Discipline | Comment |
| ICU RN | “Real-time feedback is enticing” |
| ICU RN | “It would be acceptable to ask about past psychiatric problems or treatment history” |
| Physician | “Utilize QR codes to administer survey to uncouple email address and ensure anonymity” |
| RT | “Survey was intriguing and engaging” |
| ICU RN | “Would appreciate periodic multidisciplinary debriefing sessions with physicians as part of planned interventions” |

Cognitive Pretesting:

The mean duration required to complete the survey during beta-testing was 6.97 mins +/- 2.46 mins (SD). 100% of participants reported that the questionnaire was “not overly burdensome” (5/6) or “minimally burdensome” (1/6). Participants were also “very confident” (4/6) or “confident” (2/6) their confidentiality would be maintained with this intervention. 83% (5/6) of participants stated that they were either “extremely likely” (3/6) or “likely” (2/6) to accept real-time feedback including a mental health referral if their responses suggest possible mental health distress. Only 17% (1/6) reported that they are unlikely to accept a mental health referral. All participants rated the pertinence of the questions to the healthcare workers to be high with 33% (2/6) characterizing the pertinence as excellent and 67% (4/6) as very good. Illustrative comments from the qualitative assessment are included in the table.

| Table S3 (Risk stratification and automated responses to initial screener) | |
| --- | --- |
| Risk stratification | Automated response |
| Low risk | Based on your scores, it appears that you are experiencing minimal problems with sleep, mild distress, a safe level of alcohol consumption, and are resilient in the face of challenging experiences. If you are interested in learning more about how to continue to build on your strengths, we encourage you to set up a time to meet with our MUSC Resiliency Clinician. Would you like to set up a time to meet with MUSC Resiliency Clinician |
| Mild risk | Your struggles are real and valid for a healthcare provider. Our MUSC Resiliency Clinician can support you during this difficult time. Would you like to set up a confidential meeting with MUSC Resiliency Clinician? |
| High risk | Your struggles are real and valid for a healthcare provider. Results from your scores indicate that it may be challenging to manage these struggles on your own. We strongly recommend that you set up a time to meet with our MUSC Resiliency Clinician. Would you like to set up a time to meet with MUSC Resiliency Clinician? |
| Suicidal ideation | You reported that you sometimes have thoughts that you would be better off dead, or about hurting yourself in some way. A mental health professional from the MUSC Resiliency Program will be reaching out to you within 24 hours. In the meantime, we encourage you to call the national suicide hotline (1-800-273-8255), call Trident United Way Hotline at 211, call 911, or go to the nearest emergency room if are at risk for harming yourself. |

| Table S4 (Tailored weekly check-in feedback) | |
| --- | --- |
| Level of distress | Tailored feedback response |
| Minimal distress (score <5 on K6) | It appears that you are experiencing minimal distress this week. |
| Moderate distress (score >=5- <12 on K6) | It appears that you are experiencing moderate distress this week. Our MUSC Resiliency Clinician can support you during this difficult time. Contact XXX at XXX-XXX-XXXX or XXX@musc.edu for additional tips or services |
| Severe distress (score >=13 on K6) | It appears that you are experiencing significant levels of distress this week. Our MUSC Resiliency Clinician can support you during this difficult time. Contact XXX at XXX-XXX-XXXX or XXX@musc.edu for services. |

| Table S5 (Weekly self-help tips) | |
| --- | --- |
| Week | Self-help tips |
| Week 1 | Self-awareness of your emotional and behavioral health is very important and can help you make timely decisions about what needs to be changed and when. Your reaction to stressful situations is called your stress response. This reaction typically occurs when you feel that the demands you are facing exceed your perceived ability to manage them. One of the first steps to managing stress is to pay attention to it and how it affects you. Make a list of all the stressful things you are experiencing right now and how each one impacts your body, mind, mood, and behavior. Once you have completed the list, write out things you have done that you know have been helpful and have not been helpful, as well as things you have considered, but not tried. |
| Week 2 | Self-regulation is the ability to interrupt your body's physiological response to stress while still being engaged in daily activities. This is important because it helps you become more comfortable, attentive, relaxed, resonant, and efficient even when you encounter stressors throughout the day. Two ways to consciously control your physiological response while being exposed to a stressor are breathing and muscle relaxation. Here is a very simple breathing technique that can be used for acute relaxation and improved cognitive functioning. Measured breathing. This method is done by breathing in through your nose for 4 counts and, next, by holding your breath for four counts and then exhaling through your mouth pushing as much air out as possible for four counts. You should repeat this process until your body feels relaxed and comfortable. It will also be beneficial to practice this technique even when you are not having a physical stress response. |
| Week 3 | Muscle relaxation is the second self-regulation skill. To use this skill, pay attention to how much distress and discomfort you are feeling right now. Rate it on a scale of 0-10, with 0 being none and 10 being the most distress and discomfort you have ever felt. Next, take five seconds to completely relax your muscles in your body, by first tightening them as much as you can while sitting or standing and then immediately relaxing them as if they were like wet spaghetti noodles. Repeat 5x. Then re-evaluate how much distress and discomfort you are feeling now using the same scale. Did it decrease? |
| Week 4 | Setting specific goals and clarifying your values and intentions are important practices that can help you maintain and improve your professional quality of life. It allows you to bring focus and intentionality and may help you become more deliberate in your responses towards stressors and challenges. Here are a list of common work values and principles: efficient, resilient, compassionate, healer, respectful, helpful, tolerant, teachable, precise, passionate, honest, team-player, leader, undeterred, focused, positive, selfless, graceful, relaxed, authentic, fun, respectful decisive, thorough, trustworthy, kind. Choose 3 that are important for you this week and write out 2 specific activities, behaviors, and actions that you will do to focus on these in your daily work. |
| Week 5 | A good intentionality exercise is to start your workday by acknowledging (1) what's happening at work that is distressing and out of your control (2) what strengths you bring to the team today and (3) what you hope will be true because of your work today. At the end of your workday, write out how you were able to fulfill those strengths to be there for the team today. Do this each day before and after your workday. |
| Week 6 | Please find this weekly tip, which may help foster ongoing resilience as you manage daily work stressors. Perceptual maturation is an important resilience skill that focuses on evolving your perception of yourself more so than your environment. State these affirmations out loud daily: - "There are no demands on me. I am always able to choose my reaction and response." - "I choose to do all work-related tasks, especially the undesirable ones because I want to live my values, not because someone is saying I have to." - "I have lots of strengths and even though this day might be challenging, I am going to get through this." - "I do my best and it is always good enough for today." - "I will maintain my integrity." - "I am in no danger when the system wants more from me than I can give." |
| Week 7 | We tend to develop negativity bias by looking for, reacting to, and storing negative information before we look for, react to and store positive information. You can overcome your negativity bias and look for positive information first which could increase your chances of obtaining goals and feeling happy. For the next 21 days do the following things: -Write down 3 things (small or big) that you are grateful for each day -Write for 2 minutes before going to bed describing one positive experience you have over the past 24 hours at work. -Exercise for 10 minutes a day. This trains your brain to believe your behavior matters. -Meditate for 2 minutes a day by just focusing on one thing during that time and connecting to your senses. -Write one quick email or text at the start of your workday expressing appreciation or praising a member of your team. |
| Week 8 | Building connection and support to people who feel "safe" to you is useful in being your eyes and ears. They can assist you in monitoring yourself and notifying you when you begin to show signs of stress. You give these individuals power to "call you out" in a loving and supportive way; hold you accountable for your behaviors and actions and to facilitate the safe resolution of your accumulated stress responses. So, identify 3 or more co-workers to serve as a support for you. They need to be educated by you in how to best help you and listen to you without judgment. |
| Week 9 | Self-Care and Revitalization is about providing yourself with the fuel to power yourself through your challenging day and fueling yourself at the conclusion of your day to repair and restore yourself to keep performing optimally. Here are a few suggestions to build up physical energy: -Rest -Hydrate- drink a glass a water first thing in morning and throughout the day. -Eat mindfully -Stretch your body daily |
| Week 10 | Self-Care and Revitalization is about providing yourself with the fuel to power yourself through your challenging day and fueling yourself at the conclusion of your day to repair and restore yourself to keep performing optimally. Here are some activities you can do to build your mental energy. - Learn/master a new skill or hobby - Reduce multi-tasking - Minimize distractions - Build expertise in an area of interest to you |
| Week 11 | Self-Care and Revitalization is about providing yourself with the fuel to power yourself through your challenging day and fueling yourself at the conclusion of your day to repair and restore yourself to keep performing optimally. Here are some activities to build your emotional energy. • Reduce exposure to negative people • Evaluate your expectations • Look for small joys • Get closure by finishing things and conversations that need to be finished |

| Week 12 | Self-Care and Revitalization is about providing yourself with the fuel to power yourself through your challenging day and fueling yourself at the conclusion of your day to repair and restore yourself to keep performing optimally. Here are some ways to build your spiritual energy: • Be clear about what you value by listing out the most important things in your life • Get to know your character strengths (viacharacter.org) and build one each week • Seek feedback regularly about how you are showing up to other's lives |
| --- | --- |

| Table S6 (Initial Email wording) | |
| --- | --- |
| Initial Email wording | MUSC Resiliency Program along with an interdisciplinary team of ICU providers developed a new brief screening and resource tool to assist you in understanding your stress and wellbeing, provide some recommendations, and connect you to resources.  As part of this program, you will be asked to complete a brief survey (5-7 minutes) of common difficulties of healthcare team members with real-time feedback and tailored recommendations monthly for 3 months. You will be provided with a $10 Amazon gift card for your first survey, and a $10 Amazon gift card after completing the 90-day survey as appreciation of your time. In addition, you can receive weekly very brief (1 min) screening updates and wellbeing tips via text message to help you stay in tune with your own wellbeing. Of note, this is not a research study but rather an opportunity for ongoing screening, feedback, and connection to resources. All information obtained will be completely confidential. |
